# Small Incision Lenticule Extraction (SMILE) versus Femtosecond Laser-Assisted *In Situ* Keratomileusis (FS-LASIK) for Myopia: A Systematic Review and Meta-Analysis

**DOI:** 10.1371/journal.pone.0158176

**Published:** 2016-07-01

**Authors:** Zeren Shen, Keda Shi, Yinhui Yu, Xiaoning Yu, Yuchen Lin, Ke Yao

**Affiliations:** 1 Eye Center, Second Affiliated Hospital, School of Medicine, Zhejiang University, Hangzhou, China; 2 Department of Gastroenterology, First Affiliated Hospital, School of Medicine, Zhejiang University, Hangzhou, China; University of Florence, ITALY

## Abstract

**Purpose:**

The goal of this study was to compare small incision lenticule extraction (SMILE) with femtosecond laser-assisted in situ keratomileusis (FS-LASIK) for treating myopia.

**Methods:**

The CENTRAL, EMBASE, PubMed databases and a Chinese database (SinoMed) were searched in May of 2016. Twelve studies with 1,076 eyes, which included three randomized controlled trials (RCTs) and nine cohorts, met our inclusion criteria. The overall quality of evidence was evaluated using the Grading of Recommendations Assessment, Development and Evaluation (GRADE) working group framework. Data were extracted and analysed at three to six months postoperatively. Primary outcome measures included a loss of one or more lines of best spectacle corrected visual acuity (BSCVA), uncorrected visual acuity (UCVA) of 20/20 or better, mean logMAR UCVA, postoperative mean spherical equivalent (SE) and postoperative refraction within ±1.0 D of the target refraction. Secondary outcome measures included ocular surface disease index (OSDI), tear breakup time (TBUT) and Schirmer’s 1 test (S1T) as dry eye parameters, along with corneal sensitivity.

**Results:**

The overall quality of evidence was considered to be low to very low. Pooled results revealed no significant differences between the two groups with regard to a loss of one or more lines in the BSCVA (OR 1.71; 95% CI: 0.81, 3.63; *P* = 0.16), UCVA of 20/20 or better (OR 0.71; 95% CI: 0.44, 1.15; *P* = 0.16), logMAR UCVA (MD 0.00; 95% CI: -0.03, 0.04; *P* = 0.87), postoperative refractive SE (MD -0.00; 95% CI: -0.05, 0.05; *P* = 0.97) or postoperative refraction within ±1.0 D of the target refraction (OR 0.78; 95% CI: 0.22, 2.77; *P* = 0.70) within six months postoperatively. The pooled analysis also indicated that the FS-LASIK group suffered more severely from dry eye symptoms (OSDI; MD -6.68; 95% CI: -11.76, -2.00; *P* = 0.006) and lower corneal sensitivity (MD 12.40; 95% CI: 10.23, 14.56; *P* < 0.00001) at six months postoperatively.

**Conclusions:**

In conclusion, both FS-LASIK and SMILE are safe, effective and predictable surgical options for treating myopia. However, dry eye symptoms and loss of corneal sensitivity may occur less frequently after SMILE than after FS-LASIK.

## Introduction

Laser-assisted in situ keratomileusis (LASIK) has been the standard refractive surgery used for treating myopia since the 1990s[1]. One of the critical steps in this procedure is the creation of a corneal flap[2], which is followed by corneal ablation using a separate excimer laser. This corneal flap is traditionally created by mechanical microkeratomes (MK)[3], and the application of femtosecond laser increases predictability of flap depth, allowing LASIK surgery to be safer and more precise.

With the introduction of the femtosecond laser (VisuMax, Carl Zeiss Meditec AG) in 2006, a new method of intrastromal keratomileusis, small incision lenticule extraction (SMILE), emerged[4]. SMILE is a novel form of ‘flapless’ surgery, where the lenticule is extracted through a much smaller corneal incision[5].

SMILE seems to be an option when refractive surgery is planned[6], and recent studies have reported the benefits of SMILE over FS-LASIK[7,8]. There were also conflicting reports about the postoperative visual recovery and corneal stability of these two procedures[9–11]. Thus, the aim of present study was to review in greater depth the available studies for understanding the differences of safety, efficacy and predictability between SMILE and FS-LASIK. A meta-analysis of the existing randomized controlled trials (RCTs) and cohorts using SMILE and FS-LASIK to correct myopia was performed.

## Materials and Methods

A systematic review and meta-analysis were performed in accordance with the Preferred Reporting Items for Systematic Reviews and Meta-Analyses (PRISMA) and Meta-analysis of Observational Studies in Epidemiology (MOOSE) guidelines[12, 13].

### Search strategy

Two reviewers independently searched the PubMed, EMBASE, Cochrane Central Register of Controlled Trials (CENTRAL) and a Chinese database (SinoMed) for records that compare SMILE and LASIK for treating myopia. The search terms were composed of myopia (e.g. myopia, shortsight and nearsighted), LASIK (e.g. LASIK and Keratomileusis, Laser In Situ) and SMILE (e.g. SMILE, lenticule extraction). The search process of PubMed was showed in S1 Appendix. No date or language restrictions in the electronic search for the trials were used, and the last search was run on May 4, 2016. The titles and abstracts were independently screened by two reviewers; then, the potentially relevant reports were assessed as complete manuscripts. Discrepancies between the reviewers were resolved by discussion.

### Inclusion and exclusion criteria

The following selection criteria were used to identify the studies for inclusion in this meta-analysis: 1) original papers which reported independent data, 2) adults with stable myopia or myopic astigmatism, and the absence of systemic or localized ocular disease, and 3) the use of standard surgical techniques (SMILE and FS-LASIK). Abstracts, case-reports, reviews, letters, comments non-comparative studies and non-human investigations were excluded. When multiple investigations were reported by the same team from the same institution, only the latest or the studies with the largest data set was included. Articles without outcomes of interest were excluded from this review.

### Outcome measures

Data were extracted and analysed at three to six months postoperatively. The primary outcome measures were postoperative safety, efficacy and predictability at the end of the follow-up, while the safety measure was a loss of one or more lines of best spectacle corrected visual acuity (BSCVA). The efficacy measures included the percentage of eyes with an uncorrected visual acuity (UCVA) of 20/20 or better and the mean logMAR UCVA. The postoperative mean spherical equivalent (SE) and the percentage of eyes within ±1.0 D of the target refraction were the predictability measures. The dry eye parameters, including ocular surface disease index (OSDI), tear breakup time (TBUT) and Schirmer’s 1 test (S1T), along with corneal sensitivity were also reviewed as secondary outcome measures. Overall, at least one of the primary outcome measures was required in the included studies.

### Data extraction and quality assessment

The data extraction and quality assessment were independently completed by two reviewers, and the following information was extracted from each study: first author, year of publication, study design, location, language, number of eyes enrolled, age of patients, degree of myopia, laser platform, duration of follow-up and outcome data. Those studies without all of the data points could be included, and the authors were contacted to provide more information when necessary. Five authors were contacted and one responded[14].

The risk of bias for the RCTs was evaluated using the Cochrane Risk of Bias Tool[15], while the Newcastle-Ottawa Scale (NOS) was adopted to assess each cohort[16]. The overall quality of evidence was evaluated using the Grading of Recommendations Assessment, Development and Evaluation (GRADE) working group framework.

### Statistical analysis

Meta-analysis was performed for the comparisons of outcomes. Additionally, an odds ratios (OR) and the corresponding 95% confidence interval (CI) were calculated for the dichotomous outcomes. For the continuous measures, the mean difference (MD) and the corresponding 95% CI were used, and a *P* < 0.05 was considered to be statistically significant difference. When the P-value for heterogeneity is < 0.10 or I^2^ is > 50%, substantial heterogeneity was detected. The fixed effect model (FEM) was used when no heterogeneity was observed throughout included studies. Otherwise, the random effect model (REM) was used[17,18].

Subgroup analyses were performed on the primary outcomes with regard to the study design (RCTs versus cohorts) and region (Asia versus Europe). In addition, a sensitivity analysis was performed to evaluate the robustness of the results, and each study in the meta-analysis was excluded in turn to investigate the influence of the individual studies on the pooled estimates, which was called a ‘leave-one-out’ analysis. Publication bias was estimated using Begg’s and Egger’s tests[19,20], and the statistical analyses were performed using RevMan software (version 5.3; Cochrane Collaboration, Oxford, United Kingdom) and STATA (version 12.0; Stata Corporation, College Station, Texas, USA).

## Results

### Search results

**Fig 1:**
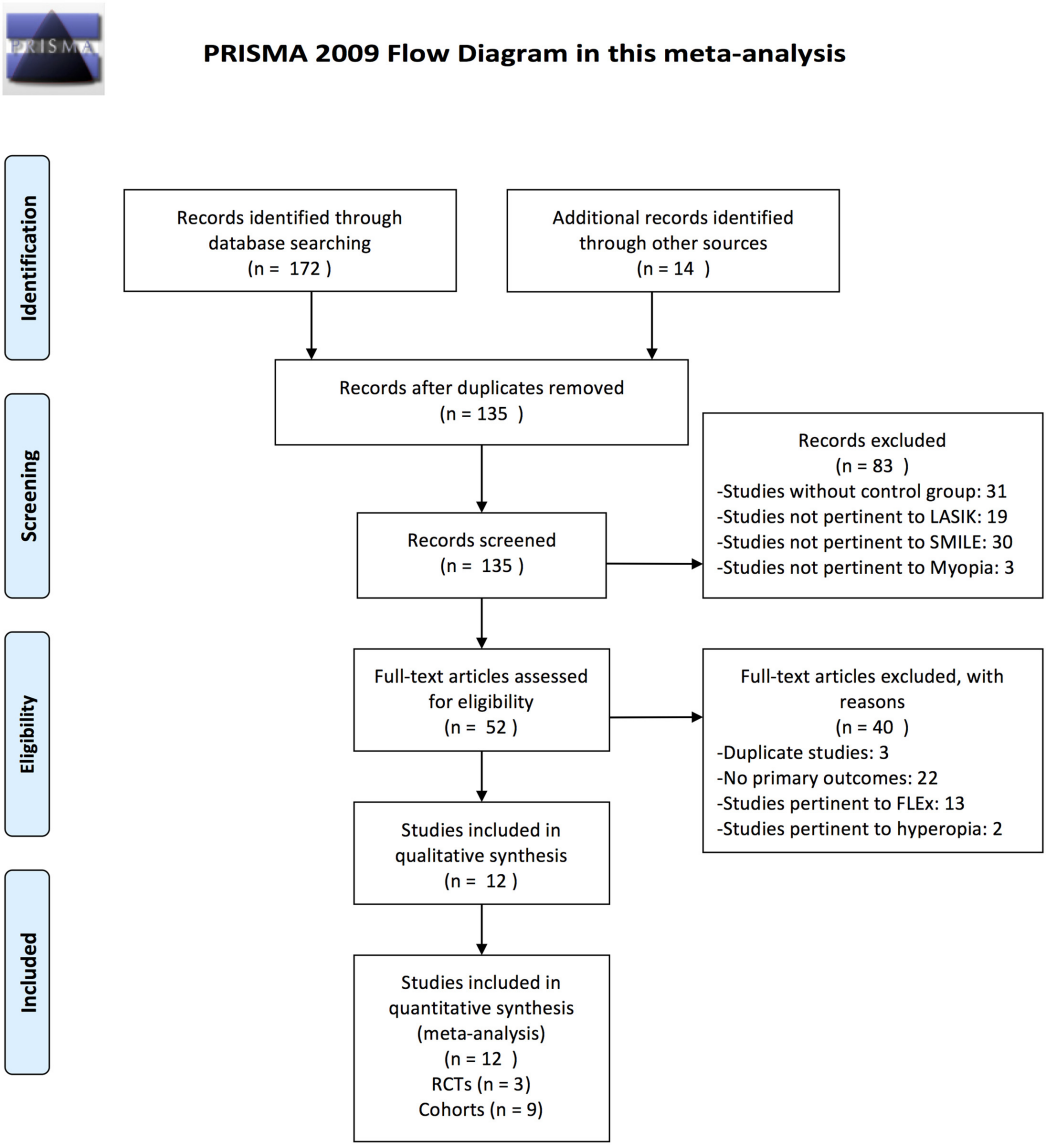
Flow chart showing selection of articles. LASIK = laser in situ keratomileusis; SMILE = small incision lenticule extraction; FLEx = femtosecond lenticule extraction; RCTs = randomized controlled trials. *From*: Moher D, Liberati A, Tetzlaff J, Altman DG, The PRISMA Group (2009). *P*referred *R*eporting *I*tems for *S*ystematic Reviews and *M*eta-*A*nalyses: The PRISMA Statement. PLoS Med 6(7): e1000097. doi:10.1371/journal.pmed1000097 For more information, visit www.prisma-statement.org.

The electronic database searches identified 186 citations, 134 of which were excluded after the initial search and screening of the titles and abstracts. After further consideration of the remaining 52 articles, 40 studies were excluded for following reasons: three studies reported the duplicate data, 22 studies did not provide the primary data identified in this study, 13 studies were pertinent to the FLEx procedure instead of the SMILE procedure, and 2 studies compared SMILE and FS-LASIK for treating hyperopia rather than treating myopia. Finally, three RCTs[7,21,22] and nine cohorts[9,10,14,23–28] were included in this meta-analysis. A flow diagram showing the search and selection process is provided in Fig 1.

### Study characteristics

A total of 1076 eyes (567 assigned to the SMILE group and 509 assigned to the FS-LASIK group) in twelve included studies were enrolled in this research. Of the twelve selected studies, nine were conducted in China[10,14,21–24,26–28], including two published in Chinese[23,24], one was conducted in France[9], one was conducted in India[7] and one was conducted in Germany[25]. The main characteristics of the included studies are described in Table 1.

**Table 1:** Characteristics of Studies Included in the Meta-analysis Comparing the SMILE and FS-LASIK. SMILE = small incision lenticule extraction, FS-LASIK = femtosecond laser-assisted LASIK, SE = spherical equivalent.

					SMILE				FS-LASIK				
Study	Year	Design	Location	Language	Eyes (n)	Age (yrs)	Preop Mean SE (D)	Femtosecond laser platform	Eyes (n)	Age (yrs)	Preop Mean SE (D)	Femtosecond laser platform and Eximer laser platfrom	Follow-up (mo)
Chan et al[14]	2015	Cohort	China	English	54	32.6 ± 9.4	-5.23 ± 1.96	VisuMax FS	57	31.3 ± 8.1	-5.82 ± 2.60	IntraLase FS and Wavelight Alleggretto Eye-Q	3
Denoyer et al[9]	2015	Cohort	France	English	30	31.1 ± 4.7	-4.65 ± 2.38	VisuMax FS	30	32.2 ± 7.5	-4.42 ± 1.78	IntraLase FS and Wavelight Alleggretto Eye-Q	6
Ganesh and Gupta[7]	2014	Randomized	India	English	50	27.4 ± 5.6	-4.95 ± 2.09	VisuMax FS	50	27.4 ± 5.6*	-3.54 ± 2.26	IntraLase FS and Schwind Amaris	3
Hu et al[23]	2013	Cohort	China	Chinese	83	25.9 ± 6.7	-4.91 ± 1.29	VisuMax FS	94	23.3 ± 5.3	-6.26 ± 2.33	VisuMax FS and Wavelight Alleggretto Eye-Q	3
Li et al[10]	2013	Cohort	China	English	38	28.2 ± 7.0	-6.68 ± 1.34	VisuMax FS	33	27.3 ± 6.6	-7.96 ± 2.61	VisuMax FS and Meditec Mel-80	6
Li et al[24]	2014	Cohort	China	Chinese	22	23.5 ± 3.5	-4.91 ± 0.90	VisuMax FS	43	28.1 ± 6.9	-6.04 ± 1.91	VisuMax FS and Wavelight Alleggretto Eye-Q	3
Lin et al[21]	2014	Randomized	China	English	60	25.9 ± 6.4	-5.13 ± 1.75	VisuMax FS	51	24.8 ± 6.2	-5.58 ± 2.41	VisuMax FS and Meditec Mel-80	3
Liu et al[22]	2016	Randomized	China	English	113	25.0 ± 5.0	-5.22 ± 1.70	VisuMax FS	84	24.0 ± 5.0	-5.18 ± 1.93	VisuMax FS and Wavelight Alleggretto Eye-Q	6
Sefat et al[25]	2015	Cohort	Germany	English	43	36.6 ± 7.7	-3.81 ± 0.95	VisuMax FS	26	36.2 ± 6.7	-3.65 ± 1.12	VisuMax FS and Meditec Mel-80	3
Shen et al[26]	2014	Cohort	China	English	17	27.1 ± 6.8	-6.48 ± 1.22	VisuMax FS	17	29.5 ± 7.4	-8.71 ± 2.02	VisuMax FS and Meditec Mel-80	3
Xia et al[27]	2016	Cohort	China	English	69	25.2 ± 4.4	-5.04 ± 2.32	VisuMax FS	59	23.7 ± 3.9	-5.13 ± 1.36	VisuMax FS and Wavelight Alleggretto Eye-Q	6
Zhang et al[28]	2016	Cohort	China	English	42	22.0 ± 5.0	-5.67 ± 1.31	VisuMax FS	22	24.0 ± 5.0	-5.21 ± 2.43	VisuMax FS and Abbott Star S4	3

* The mean age of SMILE and FS-LASIK groups, no separate data provided.

### Quality assessment

The risk-of-bias assessment of the included RCTs[7,21,22] is presented in Figures A and B in S1 File. Ganesh and Gupta’s study[7] generated an adequately randomized sequence; however, the patients were randomly allocated into one of two treatment groups per their own choice in Lin et al.’s study[21]; In addition, the randomization method was unknown in Liu et al.’s study[22]. Moreover, allocation concealment was not mentioned in any study. Whether they were conducted in a blinded fashion is unknown, but presumably this was not done because the two procedures are inherently different and the participants would know which procedure they were undergoing. There was no loss of follow-up in any of the studies, and all of them were free of reporting bias or any other bias. The NOS system was used to assess the quality of the included cohorts[9,10,14,23–28]. For selection, no selection bias was found in each study. For comparability, all studies have controlled for the most important factors. For outcome, six studies had only three-months follow-up[14,23,24–26,28]. All of the cohorts rated total scores of more than five, indicating a low risk of bias (S1 Table).

### Primary outcome criteria

#### Loss of one or more lines of BSCVA

**Fig 2:**
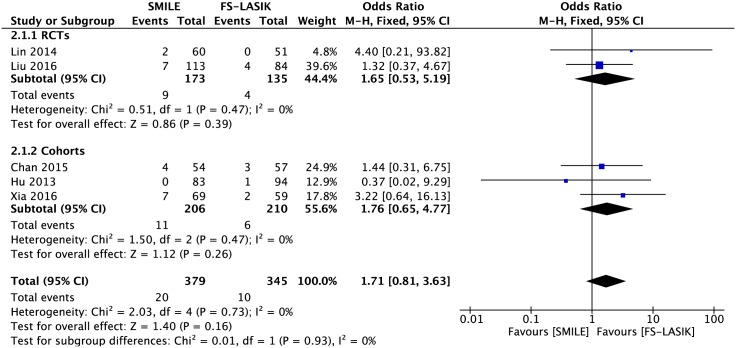
Forest plot showing the odds ratio (OR) of proportion of eyes that lost of one or more lines of best spectacle corrected visual acuity (BSCVA) comparing small incision lenticule extraction (SMILE) with femtosecond laser-assisted LASIK (FS-LASIK) within six months postoperatively. The diamonds represent the summary estimates of all five studies or the subgroup analysis of two RCTs and three cohorts.

Seven studies reported data for the percentage of eyes that lost one or more lines[7,14,21–23,27,28]. No patient lost one or more lines in two studies[7,28], and an examination of the forest plot demonstrated no significant difference between the two groups in the remaining five studies (OR 1.71; 95% CI: 0.81, 3.63; *P* = 0.16; Fig 2)[14,21–23,27]. Given the wide CI, absolute effect was applied, and no significant difference between groups was detected (RD 0.02; 95% CI: -0.01, 0.05; *P* = 0.15).

#### UCVA of 20/20 or better

**Fig 3:**
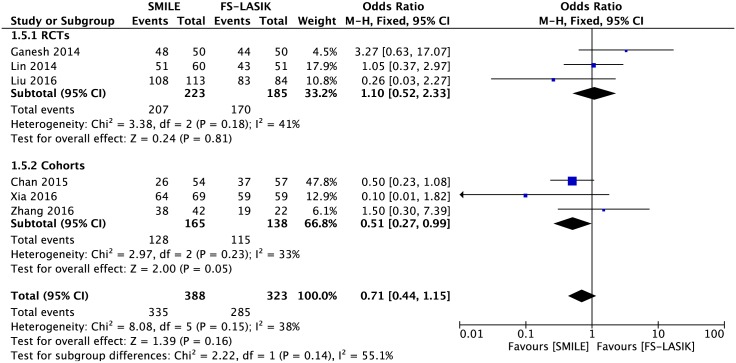
Forest plot showing the odds ratio (OR) of proportion of eyes with uncorrected visual acuity (UCVA) 20/20 or better comparing small incision lenticule extraction (SMILE) with femtosecond laser-assisted LASIK (FS-LASIK) within six months postoperatively. The diamonds represent the summary estimates of all six studies or the subgroup analysis of three RCTs and three cohorts.

At the end of the follow-up, the results of six studies presented no significant difference between the two groups in achieving a UCVA of 20/20 or better (OR 0.71; 95% CI: 0.44, 1.15; *P* = 0.16; Fig 3)[7,14,21,22,27,28]. After excluding the study by Ganesh and Gupta[7] in the sensitivity analysis, the combined result revealed that more eyes achieved a UCVA of 20/20 or better in the FS-LASIK group (OR 0.59; 95% CI: 0.35, 0.99; *P* = 0.05; S2 Table).

#### UCVA (logMAR)

**Fig 4:**
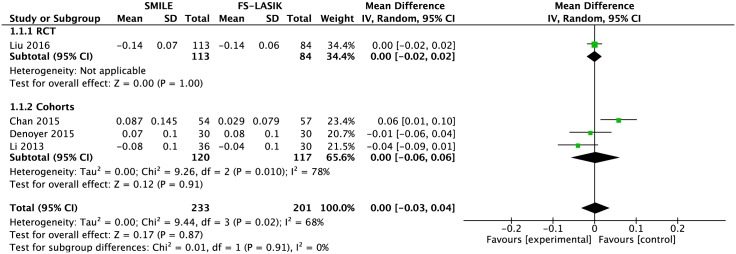
Forest plot showing the mean difference (MD) of uncorrected visual acuity (UCVA; logMAR) comparing small incision lenticule extraction (SMILE) with femtosecond laser-assisted LASIK (FS-LASIK) within six months postoperatively. The diamonds represent the summary estimates of all four studies or the subgroup analysis of one RCT and three cohorts.

Four studies compared the UCVA outcomes between the SMILE and FS-LASIK groups[9,10,14,22]. An examination of the forest plot showed no significant difference between the two groups in the UCVA (MD 0.00; 95% CI: -0.03, 0.04; *P* = 0.87; Fig 4). An evident heterogeneity was detected among the study results (I^2^ = 68%). Sensitivity analysis revealed that the Chan et al.’s study is the source of the statistical heterogeneity[14]. When this outlier study is removed, there was no evidence of heterogeneity in the remaining studies (I^2^ = 14%; S2 Table). However, the results remained unchanged (MD -0.01; 95% CI: -0.03, 0.01; *P* = 0.46; S2 Table).

#### Postoperative refractive SE

**Fig 5:**
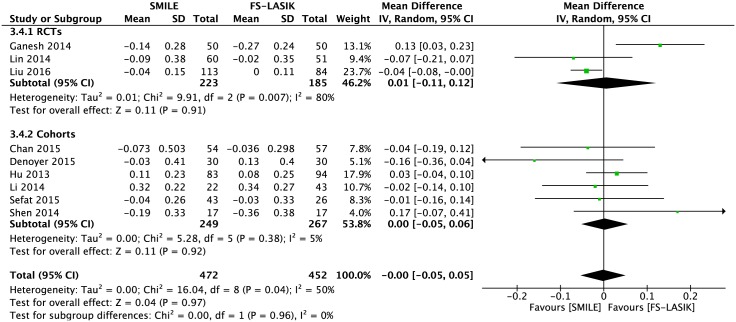
Forest plot showing the mean difference (MD) of postoperative refractive spherical equivalent (SE) comparing small incision lenticule extraction (SMILE) with femtosecond laser-assisted LASIK (FS-LASIK) within six months postoperatively. The diamonds represent the summary estimates of all nine studies or the subgroup analysis of three RCTs and six cohorts.

Nine publications reported the postoperative refractive SE (MD -0.00; 95% CI: -0.05, 0.05; *P* = 0.97; Fig 5) with no significant difference[7,9,14,21–26]. The exclusion of Ganesh and Gupta’s study reduced the heterogeneity (I^2^ from 50% to 9%) [7], but the results remained unchanged (MD -0.02; 95% CI: -0.06, 0.01; *P* = 0.18; S2 Table).

#### Postoperative refraction within ±1.0 D of the target refraction

**Fig 6:**
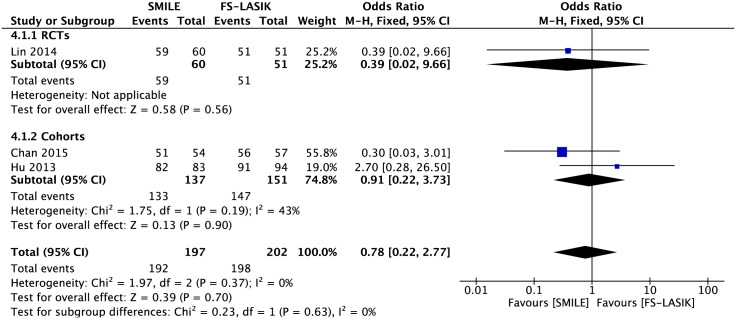
Forest plot showing the odds ratio (OR) of proportion of eyes within ±1.0 D of target refraction comparing small incision lenticule extraction (SMILE) with femtosecond laser-assisted LASIK (FS-LASIK) within six months postoperatively. The diamonds represent the summary estimates of all three studies or the subgroup analysis of one RCT and two cohorts.

Data were collected from four studies[14,21–23], and the forest plot for this outcome showed no significant difference between the two surgical procedures (OR 0.78; 95% CI: 0.22, 2.77; *P* = 0.70; Fig 6). All eyes achieved the postoperative refraction within ±1.0 D of the target refraction in Liu et al.’s study[22].

### Secondary outcome criteria

#### Dry eye symptoms

Of the twelve included studies, four mentioned dry eye postoperatively[7,9,10,27]. In Ganesh and Gupta’s study[7], reductions in Schirmer’s I and II tests and the TBUT were seen in both groups at three months. These postoperative values were significantly lower in the FS-LASIK group than in the SMILE group[7]. In the other three studies[9,10,27], data from the OSDI, TBUT and S1T at six months were available; thus, a meta-analysis was conducted for these comparisons (S1 Fig). The results showed that as compared with the SMILE group, the OSDI was significantly impaired in the FS-LASIK group (MD -6.68; 95% CI: -11.76, -2.00; *P* = 0.006; S1 Fig), leading to the severer dry eye symptoms.

#### Loss of corneal sensitivity

Three studies reported the corneal sensitivity of the SMILE and FS-LASIK groups[9,10,27]. The data from Li et al.[10] and Xia et al.[27] were available for meta-analysis. An examination of the forest plot showed that the corneal sensitivity in the SMILE group was higher than that in the FS-LASIK group (MD 12.40; 95% CI: 10.23, 14.56; *P* < 0.00001; S2 Fig).

### Quality evaluation

The overall quality of the meta-analysis is shown in Table 2. The assessment was considered to be of low to very low quality. Study design was the main reason to downgrade the overall quality of evidence, as the GRADE group suggested. Moreover, high heterogeneity, a limited number of participants and wide CIs downgraded the quality of outcomes respectively.

**Table 2:** Summary of Findings.

Outcome	Anticipated absolute effects (95% CI)		Relative effect (95% CI)	№ of participants (studies)	Quality of the evidence (GRADE)	Comments
	Risk with FS-LASIK	Risk with SMILE*				
**Loss of one or more lines of BSCVA** *follow up*: *within six months*	29 per 1,000	49 per 1,000 (24 to 98)	OR 1.71 (0.81 to 3.63)	724 (2 RCTs and 3 cohorts)	⨁◯◯◯ VERY LOW ^1^^,^^2^	30 events. CI: 0.81 to 3.63.
**UCVA of 20/20 or better** *follow up*: *within six months*	882 per 1,000	842 per 1,000 (767 to 896)	OR 0.71 (0.44 to 1.15)	711 (3 RCTs and 3 cohorts)	⨁⨁◯◯ LOW^1^	Observational studies were considered to be of a low grade.
**UCVA (logMAR)** *follow up*: *within six months*	The mean UCVA (logMAR) was 0	The mean UCVA (logMAR) in the intervention group was 0 (0.03 fewer to 0.04 more)	-	434 (1 RCT and 3 cohorts)	⨁◯◯◯ VERY LOW^1^^,^^3^	I^2^ = 68%
**Postoperative refractive SE** *follow up*: *within six months*	The mean postoperative refractive SE was 0	The mean postoperative refractive SE in the intervention group was 0 (0.05 fewer to 0.05 more)	-	924 (3 RCTs and 6 cohorts)	⨁◯◯◯ VERY LOW^1^^,^^3^	I^2^ = 50%
**Postoperative refraction within ±1.0 D of target refraction** *follow up*: *within six months*	980 per 1,000	975 per 1,000 (916 to 993)	OR 0.78 (0.22 to 2.77)	399 (1 RCT and 2 cohorts)	⨁◯◯◯ VERY LOW ^1^^,^^2^	399 participants. CI: 0.22 to 2.77.
**OSDI** *follow up*: *at six months*	The mean OSDI was 0	The mean OSDI in the intervention group was 6.88 fewer (11.76 fewer to 2 fewer)	-	254 (3 cohorts) ^1^	⨁◯◯◯ VERY LOW ^1^^,^^2^^,^^3^	254 participants. CI: -11.76 to -2.00. I^2^ = 70%
**Corneal sensitivity** *follow up*: *at six months*	The mean corneal sensitivity was 0	The mean corneal sensitivity in the intervention group was 12.4 more (10.23 more to 14.56 more)	-	194 (2 cohorts) ^1^	⨁◯◯◯ VERY LOW ^1^^,^^2^	194 participants. CI: 10.23 to 14.56.

*The risk in the intervention group (and its 95% confidence interval) is based on the assumed risk in the comparison group and the **relative effect** of the intervention (and its 95% CI). **CI:** Confidence interval; **OR:** Odds ratio; **MD:** Mean difference; **RCTs:** randomized controlled trials; **SMILE:** small incision lenticule extraction; **FS-LASIK:** femtosecond laser-assisted LASIK; **BSCVA:** best spectacle corrected visual acuity; **UCVA:** uncorrected visual acuity; **SE:** spherical equivalent; **OSDI:** ocular surface disease index.

^1^ Observational studies were considered to be of a low grade.

^2^ Few participants or events, wide CIs.

^3^ High heterogeneity.

### Publication bias

The Begg’s test (*P* = 0.296 to 1.000) and Egger’s test (*P* = 0.479 to 0.965) were applied to all of the primary outcomes, and did not reveal any publication bias.

### Sensitivity analysis

The results of the leave-one-out analysis on the majority of the outcomes showed that all exclusions did not alter the results of the previous analyses (S2 Table). In the UCVA outcome, the pooled result showed that more eyes achieved a UCVA of 20/20 or better in the FS-LASIK group after excluding the study by Ganesh and Gupta[7]. In the logMAR UCVA outcome, there was no significant heterogeneity (I^2^ = 14%) among the remaining studies after excluding the study by Chan et al.[14]. And in the postoperative refractive SE outcome, the heterogeneity was reduced (I^2^ from 50% to 9%) after excluding Ganesh and Gupta’s study.

### Subgroup Analysis

Subgroup analyses were performed on the primary outcomes with regard to the study design (RCTs versus cohorts) and region (Asia versus Europe) (Tables 3 and 4). More eyes in the FS-LASIK group than the SMILE group achieved a UCVA of 20/20 or better in cohorts, but there was no significant difference between the two groups in RCTs, which was consistent with the combined result (Fig 3, Table 3). There was no heterogeneity between the subgroups regarding study design or region in the remaining outcomes (I^2^ = 0).

**Table 3:** Subgroup Analyses on Study Design. BSCVA = best spectacle corrected visual acuity, UCVA = uncorrected visual acuity, SE = spherical equivalent, RCT = randomized controlled trials, OR = odds ratio, MD = mean difference, CI = confidence interval, I^2^ = extent of inconsistency.

Study design (RCTs versus cohorts)	Studies	Eyes (n)	Effect measure		Test for subgroup differences	
			OR or MD (95% CI)	I^2^	I^2^	Chi^2^ P value
Loss of one or more lines of BSCVA			OR 1.71 [0.81, 3.63]	0%	0%	0.93
RCTs	2	308	OR 1.65 [0.53, 5.19]	0%		
Cohorts	3	416	OR 1.76 [0.65, 4.77]	0%		
UCVA of 20/20 or better			OR 0.71 [0.44, 1.15]	38%	55.1%	0.14
RCTs	3	408	OR 1.10 [0.52, 2.33]	41%		
Cohorts	3	303	OR 0.51 [0.27, 0.99]	33%		
UCVA (log MAR)			MD 0.00 [-0.03, 0.04]	68%	0%	0.91
RCT	1	197	MD 0.00 [-0.02, 0.02]	-		
Cohorts	3	237	MD 0.00 [-0.06, 0.06]	78%		
Postoperative refractive SE			MD -0.00 [-0.05, 0.05]	50%	0%	0.96
RCTs	3	408	MD 0.01 [-0.11, 0.12]	80%		
Cohorts	6	516	MD 0.00 [-0.05, 0.06]	5%		
Postoperative refraction within ±1.0 D of the target refraction			OR 0.78 [0.22, 2.77]	0%	0%	0.63
RCT	1	111	OR 0.39 [0.02, 9.66]	-		
Cohorts	2	288	OR 0.91 [0.22, 3.73]	43%		

**Table 4:** Subgroup Analyses on Region. BSCVA = best spectacle corrected visual acuity, UCVA = uncorrected visual acuity, SE = spherical equivalent, OR = odds ratio, MD = mean difference, CI = confidence interval, I^2^ = extent of inconsistency.

Region (Asia versus Europe)	Studies	Eyes (n)	Effect measure		Test for subgroup differences	
			OR or MD (95% CI)	I^2^	I^2^	Chi^2^ P value
UCVA (log MAR)			MD 0.00 [-0.03, 0.04]	68%	0%	0.64
Asia	3	374	MD 0.01 [-0.04, 0.05]	78%		
Europe	1	60	MD -0.01 [-0.06, 0.04]	-		
Postoperative refractive SE			MD -0.00 [-0.05, 0.05]	50%	0%	0.32
Asia	7	795	MD 0.01 [-0.05, 0.07]	57%		
Europe	2	129	MD -0.07 [-0.21, 0.08]	26%		

## Discussion

The present systematic review and meta-analysis identified three RCTs and nine cohorts investigating the effects of SMILE and FS-LASIK for the correction of myopia. In our analysis of six months follow-up, it was found that SMILE achieved similar safety, efficacy and predictability to FS-LASIK. Additionally, the incidences of postoperative dry eye symptoms and loss of corneal sensitivity in the SMILE group were lower than those in the FS-LASIK group.

Although some cohorts have recently reported comparisons of SMILE and FS-LASIK, RCTs have rarely been published. The differences in the baseline, such as age or gender, are unlikely to be significant factors contributing to the study results in refractive surgery directed at the cornea. In addition, subgroup analyses focusing on study design showed no differences between the results of RCTs and cohorts except in the UCVA outcome. Moreover, the results of the RCTs are always consistent with the combined results. Thus, it is feasible and important to summarize all of the published information, because doing so may help clinicians make the optimal decision for patients[29].

A major difficulty found in conducting the analysis was the diversity of follow-up interval variations. There is no generally accepted method for reporting the results of trials involving refractive procedures[30]. Based on previous studies[22,31,32] and our clinical experience, the parameters of efficacy, safety and predictability remain stable at three months postoperatively and beyond. Thus, the data reported at the end of the follow-up were pooled for comparison.

The results of this meta-analysis showed that both SMILE and FS-LASIK are safe, effective and predictable. In terms of safety, the examination of the forest plot revealed that the percentage of eyes losing one or more lines in the SMILE group (5.3% in average) was small and similar to that in the FS-LASIK group (2.9% in average). Moreover, two studies reported no patient losing one or more lines post-operation[7,28].

In terms of predictability, both groups achieved excellent postoperative residual error in the included studies[7,9,14,21–26]. We found no significant differences between the two groups with regard to the postoperative refractive SE and the proportion of postoperative refraction within ±1.0 D of the target refraction. In particular, Ganesh and Gupta’s study suggested that SMILE is more predictable than FS-LASIK because the creation of a flap in FS-LASIK exposes the stroma to hydration changes, leading to the inaccurate removal of the stromal tissue. However, the remaining studies showed no differences in predictability between the two groups[7], which is consistent with the combined result. The explanation for this difference may lie in the different laser platforms used. The IntraLase femtosecond laser and Schwind Amaris excimer laser were used in the FS-LASIK procedure in Ganesh and Gupta’s study. There are trials reporting that VisuMax achieved fewer complications than IntraLase[33,34]. Moreover, one meta-analysis revealed that the Abbott Star S4 and Mel-80 excimer platforms are more effective than the Schwind Amaris platform[35]. However, others reported no significant differences between VisuMax and IntraLase[36,37,38]. A prospective case series reported no differences in the efficacy between the Schwind Amaris and Wavelight Alleggretto Eye-Q excimer platforms[39]. The influence of laser platform could not be further explored due to the limited number of studies available.

In terms of efficacy, no significant differences were detected between the two groups in the UCVA outcomes. Nevertheless, the I^2^ value in the UCVA in logMAR outcome indicates significant between-study heterogeneity. Sensitivity analysis revealed that the Chan et al.’s study[14] is the source of statistical heterogeneity in the meta-analysis for the logMAR UCVA. There was no evidence of heterogeneity in the three remaining studies after excluding Chan et al.’s study, but the exclusion did not alter the result of the previous analysis. Chan et al.’s study includes only patients with myopic astigmatism, and it supports FS-LASIK as an optimization in treating patients with myopic astigmatism. However, Zhang et al.’s study, which also includes only myopic astigmatic patients, reports no difference in efficacy between FS-LASIK and SMILE. Thus, the heterogeneity may arise from the limited number of studies and external factors. For this reason, the results in this analysis were pooled using a random effects model. Moreover, sensitivity analysis did not alter most of the results of the primary analyses, which indicates that the combined results were robust and reliable.

In consideration of visual quality, further attention should be paid to the influence of surgery on complications rather than on visual acuity alone. Awareness has been growing regarding the occurrence of dry eye symptoms and the loss of corneal sensitivity after refractive surgery, and three of the included studies compared the measurements between the two groups with regard to these complications[9,10,27]. The data showed fewer dry eye symptoms and greater corneal sensitivity in the SMILE group than in the FS-LASIK group six months after surgery. One hypothesis is that the most important factor in the pathophysiology of refractive surgery-induced dry eye symptoms and decreased corneal sensitivity is the transection of the corneal nerves that occurs during these surgeries[40]. Since flap creation severs most corneal nerves around the ring, a lower corneal nerve density and a smaller number of long fibres and secondary branching were observed in the FS-LASIK as compared to the SMILE eyes[9]. These findings provide us with a reasonable explanation. As an all-in-one femtosecond laser flapless procedure, SMILE likely minimizes the change in the shape of the cornea, maintaining biomechanical stability to the largest extent possible[21].

Our findings are similar to reviews comparing SMILE and FS-LASIK conducted by Lee et al. and Zhang et al[6,41]. However, Miao et al.’s meta-analysis reported a dissimilar finding that corneal sensitivity in the SMILE group is better than the FS-LASIK group during the first three postoperative months, but similar at six months after surgery[42]. There are only five studies included in this meta-analysis and the heterogeneity is significant among all the results.

The results of this meta-analysis should be interpreted in the context of several important limitations. First, the preoperative mean SE was statistically different between the SMILE and FS-LASIK groups in some studies[7,10,23–26], which indicates a probable imbalance between the study groups and may influence results. Because of the insufficient number of included studies, the impact of this imbalance could not be formally explored for subgroup analysis.

Second, most of the studies were from Asia. Although the subgroup analysis focusing on region revealed no significant differences between the results of Asia and Europe, the results may not be generalizable to other parts of the world.

Third, one study[25] was sponsored by Zeiss and the authors of another study[9] were consultants or board members of Alcon or Abbott Medical Optics. However, the data extracted from these two studies did not reveal any preference for any corporate connections.

Finally, there was significant statistical heterogeneity in the secondary outcomes. Those studies reporting only secondary outcomes without primary outcomes were not included in our review. Data such as high-order aberrations and satisfaction score were insufficient for the meta-analysis; thus, further meta-analyses including all of the available studies for the secondary outcomes should be performed.

In conclusion, both SMILE and FS-LASIK are safe, effective and predictable surgical options for the correction of myopia. In addition, dry eye symptoms and loss of corneal sensitivity may occur less frequently after SMILE than after FS-LASIK. However, our findings, which relied largely on data from cohorts, were considered to be of low to very low quality. This conclusion should therefore be interpreted cautiously; high-quality, adequately powered RCTs with a sufficient length of follow-up are warranted.

## Supporting Information

S1 Appendix Search strategy of PubMed. (DOCX)

S2 Appendix PRISMA-checklist in this meta-analysis. (DOCX)

S1 Fig Forest plot showing the mean difference (MD) of dry eye symptom parameters comparing small incision lenticule extraction (SMILE) with femtosecond laser-assisted LASIK (FS-LASIK) at six months postoperatively. (A) Ocular surface disease index (OSDI; 1–100). (B) Tear breakup time (TBUT; s). (C) Schirmer’s 1 test (S1T) scores (mm).
(DOCX)

S2 Fig Forest plot showing the mean difference (MD) of corneal sensitivity comparing small incision lenticule extraction (SMILE) with femtosecond laser-assisted LASIK (FS-LASIK) at six months postoperatively. (EPS)

S1 Table Risk-of-bias assessment of the observational studies (cohorts). (DOCX)

S2 Table Results of leave-one-out analysis. (DOCX)

S1 File Risk-of-bias assessment of the randomized controlled trials (RCTs). (DOCX)
