# Supplementary material for: Small Incision Lenticule Extraction (SMILE) versus Femtosecond Laser-Assisted In Situ Keratomileusis (FS-LASIK) for Myopia: A Systematic Review and Meta-Analysis
Source: PLoS One. 2016 Jul 1;11(7):e0158176. doi: 10.1371/journal.pone.0158176 (PMC4930219; doi:10.1371/journal.pone.0158176)
Supplement: S1 Appendix — (DOCX) [file pone.0158176.s001.docx]

S1 Appendix. Medline (PubMed) search strategy

#1 Myopia[Mesh]

#2 myop*[tiab]

#3 (short[tiab] OR near[tiab]) AND sight*[tiab]

#4 nearsighted*[tiab]

#5 #1 OR #2 OR #3 OR #4

#6 Keratomileusis, Laser In Situ[Mesh]

#7 Keratomileus*[tiab]

#8 LASIK [tiab]

#9 #6 OR #7 OR #8

#10 lenticule extraction[tiab]

#11 ReLEx[tiab]

#12 SMILE[tiab]

#13 #10 OR #11 OR #12

#14 #5 AND #9 AND #13
