# Supplementary material for: Small Incision Lenticule Extraction (SMILE) versus Femtosecond Laser-Assisted In Situ Keratomileusis (FS-LASIK) for Myopia: A Systematic Review and Meta-Analysis
Source: PLoS One. 2016 Jul 1;11(7):e0158176. doi: 10.1371/journal.pone.0158176 (PMC4930219; doi:10.1371/journal.pone.0158176)
Supplement: S1 Fig — (A) Ocular surface disease index (OSDI; 1–100). (B) Tear breakup time (TBUT; s). (C) Schirmer’s 1 test (S1T) scores (mm). (DOCX) [file pone.0158176.s003.docx]

A

B

C

**S1 Fig.** **Forest plot showing the mean difference (MD) of dry eye symptom parameters comparing small incision lenticule extraction (SMILE) with femtosecond laser-assisted LASIK (FS-LASIK) at 6 months postoperatively.** (A) Ocular surface disease index (OSDI; 1-100). (B) Tear breakup time (TBUT; s). (C) Schirmer’s 1 test (S1T) scores (mm).
