# Supplementary material for: Small Incision Lenticule Extraction (SMILE) versus Femtosecond Laser-Assisted In Situ Keratomileusis (FS-LASIK) for Myopia: A Systematic Review and Meta-Analysis
Source: PLoS One. 2016 Jul 1;11(7):e0158176. doi: 10.1371/journal.pone.0158176 (PMC4930219; doi:10.1371/journal.pone.0158176)
Supplement: S1 Table — (DOCX) [file pone.0158176.s005.docx]

**S1 Table.** **Risk-of-Bias Assessment of the Observational Studies (cohorts)^#^**

|  | **Selection** | | | | **Comparability** | **Outcome** | | | **Total score** |
| --- | --- | --- | --- | --- | --- | --- | --- | --- | --- |
| **Study** | **Exposed cohort** | **Nonexposed cohort** | **Ascertainment of exposure** | **Outcome of interest** |  | **Assessment of outcome** | **Length of follow-up** | **Adequacy of follow-up** |  |
| Chan 2015 | * | * | * | * | ** | * | - | * | 8 |
| Denoyer 2015 | * | * | * | * | ** | * | * | * | 9 |
| Hu 2013 | * | * | * | * | * | * | - | * | 7 |
| Li 2013 | * | * | * | * | * | * | * | * | 8 |
| Li 2014 | * | * | * | * | * | * | - | * | 7 |
| Sefat 2015 | * | * | * | * | * | * | - | * | 7 |
| Shen 2014 | * | * | * | * | * | * | - | * | 7 |
| Xia 2016 | * | * | * | * | ** | * | * | * | 9 |
| Zhang 2016 | * | * | * | * | ** | * | - | * | 8 |

^#^Risk of bias was assessed with use of the Newcastle–Ottawa Scale.

A higher overall score corresponds to a lower risk of bias; a score of five or less (out of nine) indicates a high risk of bias.
