# Supplementary material for: Small Incision Lenticule Extraction (SMILE) versus Femtosecond Laser-Assisted In Situ Keratomileusis (FS-LASIK) for Myopia: A Systematic Review and Meta-Analysis
Source: PLoS One. 2016 Jul 1;11(7):e0158176. doi: 10.1371/journal.pone.0158176 (PMC4930219; doi:10.1371/journal.pone.0158176)
Supplement: S2 Table — (DOCX) [file pone.0158176.s006.docx]

**S2 Table.** **Results of leave-one-out analysis**

| **Study excluded** | **OR or MD (95% CI)** | | |  | **Test for heterogeneity** | | |  | **Test for overall effect** | |
| --- | --- | --- | --- | --- | --- | --- | --- | --- | --- | --- |
|  | **Estimate** | **Lower** | **Up** |  | **Chi^2^** | **I^2^** | **P** |  | **Z** | **P** |
| **Loss of one or more lines of BSCVA; OR** | | | | | | | | | | |
| Chan 2015 | 1.80 | 0.76 | 4.27 |  | 1.98 | 0% | 0.58 |  | 1.34 | 0.18 |
| Hu 2013 | 1.91 | 0.87 | 4.22 |  | 1.15 | 0% | 0.77 |  | 1.61 | 0.11 |
| Lin 2014 | 1.58 | 0.72 | 3.45 |  | 1.61 | 0% | 0.66 |  | 1.15 | 0.25 |
| Liu 2016 | 1.97 | 0.77 | 5.03 |  | 1.81 | 0% | 0.61 |  | 1.42 | 0.16 |
| Xia 2016 | 1.39 | 0.58 | 3.29 |  | 1.20 | 0% | 0.75 |  | 0.74 | 0.46 |
| **UCVA of 20/20 or better; OR** | | | | | | | | | | |
| Chan 2015 | 0.90 | 0.48 | 1.69 |  | 6.31 | 37% | 0.18 |  | 0.34 | 0.74 |
| *Ganesh 2014 | 0.59 | 0.35 | 0.99 |  | 4.70 | 15% | 0.32 |  | 1.62 | 0.11 |
| Lin 2014 | 0.63 | 0.36 | 1.10 |  | 7.49 | 47% | 0.11 |  | 0.65 | 0.51 |
| Liu 2016 | 0.76 | 0.46 | 1.26 |  | 7.10 | 44% | 0.13 |  | 1.05 | 0.29 |
| Xia 2016 | 0.80 | 0.48 | 1.32 |  | 6.13 | 35% | 0.19 |  | 0.87 | 0.38 |
| Zhang 2016 | 0.66 | 0.39 | 1.10 |  | 7.24 | 45% | 0.12 |  | 1.61 | 0.11 |
| **UCVA (logMAR); MD** | | | | | | | | | | |
| ^#^Chan 2015 | -0.01 | -0.03 | 0.01 |  | 2.33 | 14% | 0.31 |  | 0.75 | 0.46 |
| Denoyer 2015 | 0.01 | -0.04 | 0.05 |  | 9.19 | 78% | 0.01 |  | 0.27 | 0.78 |
| Li 2013 | 0.01 | -0.02 | 0.05 |  | 6.21 | 68% | 0.04 |  | 0.76 | 0.45 |
| Liu 2016 | 0.00 | -0.06 | 0.06 |  | 9.26 | 78% | 0.01 |  | 0.12 | 0.91 |
| **Postoperative refractive SE; MD** | | | | | | | | | | |
| Chan 2015 | 0.00 | -0.05 | 0.06 |  | 15.96 | 56% | 0.03 |  | 0.08 | 0.94 |
| Denoyer 2015 | 0.01 | -0.04 | 0.06 |  | 14.09 | 50% | 0.05 |  | 0.27 | 0.79 |
| ^#^Ganesh 2014 | -0.02 | -0.06 | 0.01 |  | 7.67 | 9% | 0.36 |  | 1.35 | 0.18 |
| Hu 2013 | -0.01 | -0.07 | 0.05 |  | 14.19 | 51% | 0.05 |  | 0.24 | 0.81 |
| Li 2014 | 0.00 | -0.06 | 0.06 |  | 16.04 | 56% | 0.02 |  | 0.05 | 0.96 |
| Lin 2014 | 0.01 | -0.05 | 0.06 |  | 15.39 | 55% | 0.03 |  | 0.22 | 0.82 |
| Liu 2016 | 0.01 | -0.05 | 0.07 |  | 11.77 | 41% | 0.11 |  | 0.34 | 0.73 |
| Sefat 2015 | -0.00 | -0.06 | 0.06 |  | 16.04 | 56% | 0.02 |  | 0.00 | 1.00 |
| Shen 2014 | -0.01 | -0.06 | 0.04 |  | 13.71 | 49% | 0.06 |  | 0.30 | 0.76 |
| **Postoperative refraction within ±1.0 D of target refraction; OR** | | | | | | | | | | |
| Chan 2015 | 1.38 | 0.26 | 7.28 |  | 0.94 | 0% | 0.33 |  | 0.38 | 0.70 |
| Hu 2013 | 0.33 | 0.05 | 2.13 |  | 0.01 | 0% | 0.91 |  | 1.17 | 0.24 |
| Lin 2014 | 0.91 | 0.22 | 3.73 |  | 1.75 | 43% | 0.19 |  | 0.13 | 0.90 |

OR = odds ratio, MD = mean difference, CI = confidence interval, I^2^ = extent of inconsistency.

* The combined results were changed after excluding this study. ^#^There was no evidence for heterogeneity after excluding these studies.
