# Supplementary material for: Small Incision Lenticule Extraction (SMILE) versus Femtosecond Laser-Assisted In Situ Keratomileusis (FS-LASIK) for Myopia: A Systematic Review and Meta-Analysis
Source: PLoS One. 2016 Jul 1;11(7):e0158176. doi: 10.1371/journal.pone.0158176 (PMC4930219; doi:10.1371/journal.pone.0158176)
Supplement: S1 File — (DOCX) [file pone.0158176.s007.docx]

**Figure A. Risk of bias graph.**

**Figure B. Risk of bias summary.**

**S1 File (Figures A and B).** **Risk-of-bias assessment of the randomized controlled trials (RCTs).** Ganesh and Gupta’s study generated an adequately randomized sequence; however, the patients were randomly allocated into one of two treatment groups per their own choice in Lin et al.’s study; In addition, the randomization method was unknown in Liu et al.’s study. Moreover, allocation concealment was not mentioned in any study. Whether they were conducted in a blinded fashion is unknown, but presumably this was not done because the two procedures are inherently different and the participants would know which procedure they were undergoing. There was no loss of follow-up in any of the studies, and all of them were free of reporting bias or any other bias.
